# Helicobacter Pylori Negative Extranodal Zone B Cell Lymphoma Presented as a Polypoid Gastric Mass: A Case Report

**DOI:** 10.4021/wjon457w

**Published:** 2012-04-23

**Authors:** Umit Akyuz, Filiz Akyuz, Kamil Ozdil, Hasan Altun, Mine Gulluoglu, Dilek Yilmazbayhan

**Affiliations:** a Department of Gastroenterology, Yeditepe University, Turkey; b Department of Gastroenterology, Istanbul Faculty of Medicine, Istanbul University, Turkey; c Department of Gastroenterology, Umraniye Educational and Research Center , Turkey; d Department of General Surgery, Fatih Sultan Mehmet Educational and Research Center, Turkey; e Department of Pathology, Istanbul Faculty of Medicine, Istanbul University, Turkey

## To the Editor

Extranodal marginal-zone B-cell lymphoma is generally arising from mucosa and associated with Helicobacter pylori (H.pylori) in 90% of patients. It is generally found in antrum endoscopically but, it is multifocal in 30% of patients. Endoscopic findings include erosions, erythematous lesions and ulcerations [1-3]. In this report, we present an unusual case with a diagnosis of H.pylori negative extranodal marginal-zone B-cell lymphoma.

**Figure 1:**
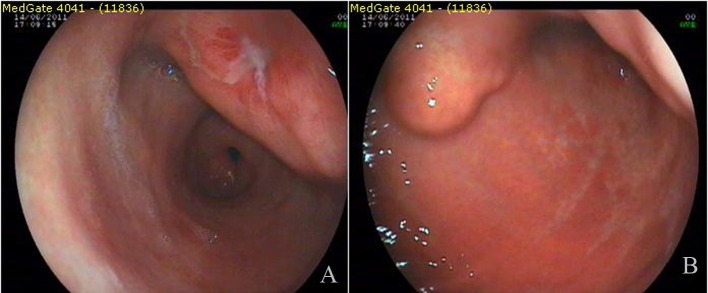
Endoscopic appearance of the lesions.

**Figure 2:**
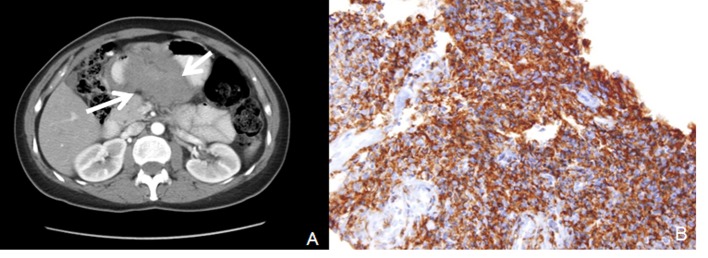
A: Computerized tomography revealed antral mass; B: CD 20 positivity by immunohistochemical study in lymphoma cells.

A 3 - 4 cm ulcerated polypoid mass in antrum and polypoid lesion with an impression of submucosal localization over the distal corpus (Fig. 1) were found in endoscopy in a female patient complaining of dyspeptic problems with an age of 54. Endoscopic biopsies revealed chronic inflammation and H.pylori was negative. First, abdominal tomography and then endoscopic ultrasonography were performed. Abdominal tomography showed a polypoid mass in the antrum with a diameter of 6 cm (Fig. 2A). Endoscopic ultrasonography yielded 5 - 6 cm transmural mass with serosal infiltration and heterogenous echogenity. Fine-needle aspiration biopsies were performed two times with a nondiagnostic material. Trucut biopsy was performed after cytologic examination yielding sample rich in lymphoid tissue. Histologic examination was reported as extranodal marginal-zone B-cell lymphoma with CD20 antigen (+) (Fig. 2B). The patient was consulted to Oncology Service for chemotherapy.

We can detect different endoscopic lesions in gastric lymphomas. Diagnostic value of fine needle aspiration biopsy in lymphoid tumors is limited.

## Abbreviations

H.pylori: Helicobacter pylori

## References

[1] Mehra M, Agarwal B (2008). Endoscopic diagnosis and staging of mucosa-associated lymphoid tissue lymphoma. Curr Opin Gastroenterol.

[2] Ruskone-Fourmestraux A, Fischbach W, Aleman BM, Boot H, Du MQ, Megraud F, Montalban C (2011). EGILS consensus report. Gastric extranodal marginal zone B-cell lymphoma of MALT. Gut.

[3] Zucca E, Bertoni F, Roggero E, Cavalli F (2000). The gastric marginal zone B-cell lymphoma of MALT type. Blood.

